# When is the right time to change therapy? An observational study of the time to response to immunosuppressive drugs in systemic lupus erythematosus

**DOI:** 10.1136/lupus-2024-001207

**Published:** 2024-07-23

**Authors:** Chiara Tani, Michele Maffi, Giancarlo Cascarano, Viola Signorini, Dina Zucchi, Marina Menchini, Chiara Stagnaro, Linda Carli, Elena Elefante, Francesco Ferro, Chiara Cardelli, Maria Laura Manca, Marta Mosca

**Affiliations:** 1 Department of Clinical and Experimental Medicine, University of Pisa, Pisa, Italy; 2 Rheumatology Unit, Azienda Ospedaliero Universitaria Pisana, Pisa, Italy; 3 Department of Medical Biotechnologies, University of Siena, Siena, Italy; 4 Department of Clinical and Experimental Medicine and Department of Mathematics, University of Pisa, Pisa, Italy

**Keywords:** systemic lupus erythematosus, biological products, therapeutics

## Abstract

**ABSTRACT:**

**Objectives:**

To analyse the response to immunosuppressants (IS) in extrarenal flares of SLE to determine the most appropriate timing during follow-up for a change in therapeutic strategy.

**Methods:**

Observational cohort study including a total of 81 patients with SLE with extrarenal flares requiring a change in IS over the period 2015–2022. Baseline clinical variables were described, and follow-up data at 1, 3, 6 and 12 months time-points were collected.

**Results:**

Among patients flaring that achieved lupus low disease activity state (LLDAS5) at 12 months of follow-up, we identified two subgroups (‘late responders’ and ‘early responders’), which showed no significant differences in demographic characteristics, baseline clinical data, cumulative dosage of glucocorticoids or type of IS. Cox model analysis revealed a significant association of a change in IS (p=0.019) and achieving LLDAS5. Contingency table analysis indicated a significant relationship (p=0.004) between IS change at 6 months and individuals achieving LLDAS5 and remission at 12 months.

**Conclusions:**

Our findings suggest that clinical improvement of extrarenal flares typically occurs within 6 months of initiating IS. This timeframe could represent an appropriate timing to evaluate the response in a treat-to-target approach in SLE.

**WHAT IS ALREADY KNOWN ON THIS TOPIC**

**WHAT THIS STUDY ADDS**

- Our findings suggest that clinical improvement of extrarenal flares typically occurs within 6 months of initiating a new immunosuppressant therapy.

**HOW THIS STUDY MIGHT AFFECT RESEARCH, PRACTICE OR POLICY**

- In a clinical practice perspective, a 6-month period from the initiation of new therapy could represent the timing to evaluate the response in a treat-to-target approach in SLE.

## Introduction

 SLE is a complex autoimmune disease impacting a spectrum of organs and systems and characterised by a dynamic relapsing-remitting pattern.^1^

Drawing inspiration from successful applications in managing various chronic disorders, the treat-to-target (T2T) approach was proposed also for SLE treatment.^2^ This strategy encompasses a systematic framework involving the selection of a defined therapeutic target, the establishment of quantifiable measures to gauge progress, the implementation of tailored interventions to attain the defined objective, scheduled time-points for re-evaluation and the adaptation of therapy if the desired outcome remains elusive.^3^ Over the following years, remission and lupus low disease activity state (LLDAS) have been defined, and their pursuit has led to improved care for patients with chronic diseases.^4 5^ The attainment of these targets is now recommended in the 2023 update of the EULAR recommendations, as well as treatment targets for lupus nephritis and timeframes within which they should be achieved, to ensure a favourable long-term outcome.^6^

However, in non-renal lupus, it is uncertain how soon the therapeutic target should be achieved and when a shift in strategy should be considered in order to avoid keeping the disease not well controlled for a long time or, alternatively, making premature changes in therapy.

Following this line of thought, the aims of our study were to describe the characteristics of extrarenal flares treated with immunosuppressive (IS) drugs, and to analyse the response to therapy over 12 months, to assess what might be the most appropriate time during follow-up for a shift in therapeutic strategy.

## Methods

This study presents an observational analysis of a monocentric cohort comprising patients with SLE in regular follow-up at the Lupus Clinic of the Rheumatology Unit, University of Pisa, Italy.

The research focused on consecutive disease flares occurring between January 2015 and July 2022 in patients who met either the 1997 revised American College of Rheumatology (ACR) criteria or the 2019 EULAR-ACR criteria for SLE.^7 8^

Patients flaring were defined using the Safety of Estrogens in Lupus Erythematosus National Assessment (SELENA)-SLE Disease Activity Index (SLEDAI) Flare Index^9 10^ and were categorised as either ‘renal’ and ‘extrarenal’ flares (musculoskeletal, mucocutaneous, constitutional, vasculitic, haematological, cardiopulmonary, neuropsychiatric). For this analysis, only extrarenal flares were considered. Besides, flares treated only with glucocorticoids (GC) and hydroxychloroquine were not included, since we wanted to focus our study solely on patients treated with the introduction of a new immunosuppressant (IS) or a therapeutic switch. To ensure data quality, only patients flaring with a minimum of 1-year follow-up and complete clinical and laboratory information extracted from clinical charts were included in this analysis.

Patients were assessed at the time of flare occurrence (baseline) and subsequently at 1, 3, 6 and 12 months. From each patient, we collected the following data: demographics (age, sex, ethnicity, body weight, disease duration), previous organ involvement, overlap with antiphospholipid syndrome defined according to the Revised Sapporo Classification Criteria^11^ and Sjögren’s syndrome defined according to the 2016 EULAR-ACR classification criteria.^12^ Additionally, we recorded the presence of hypocomplementaemia and/or antidouble stranded DNA at the time of flare, ongoing therapies at flare occurrence and any new drug prescriptions. Regarding GC treatment, we documented the number of GC pulses, if any, and the daily oral prednisone (PDN) dosage administered to treat the flare, both in milligrams and per kilogram of body weight. Furthermore, we collected data on the cumulative PDN dosage throughout the study duration and the daily dosage taken by the patient at follow-up time-points. Disease activity was assessed using the SELENA-SLEDAI^8^ and British Isles Lupus Assessment Group (BILAG) scores^13 14^ at baseline, 3, 6 and 12 months. The BILAG score was calculated solely for the most severe organ manifestation that guided the therapeutic choice. Additionally, at each time-point, the fulfilment of criteria for clinical remission (according to DORIS definition, excluding patient global assessment)^15^ and modified LLDAS5 (excluding patient global assessment and with a daily GC dosage ≤5 mg)^16^ was evaluated. Improvement in SLEDAI from baseline to month 3 was also evaluated; an improvement of at least 4 points in the SLEDAI score was considered significant. Organ damage was evaluated at baseline and 12 months using the Systemic Lupus International Collaborating Clinics/ACR-Damage Index (SDI).^17 18^

All patients consent for the processing of their clinical data on entering the Pisa Lupus cohort.

### Statistical analysis

Continuous data were presented as mean and SD or median and IQR, while for categorical variables, percentage in each category was displayed.

A Cox survival analysis to study the association between the change of IS, as a time-varying covariate, and the remission was implemented. Age and disease duration were also entered as covariates in this analysis. The analysis was repeated considering the LLDAS5 variable as outcome.

The associations between categorical variables were studied with contingency tables and generalised χ^2^ tests.

## Results

Among 444 patients with SLE regularly followed at the Pisa Lupus cohort, over the last 8 years a total of 81 extrarenal flares required a change in the IS treatment (adding or changing IS) and were included in this analysis. Demographic and clinical characteristics as well as previous organ involvement are summarised in table 1.

**Table 1:** Demographic, clinical data and ongoing treatment at study enrolment (flare occurrence)

Demographic data and clinical history	Whole cohort (n=81)
Age (year, mean±SD)	41±12.4
Disease duration (year, mean±SD)	9.9±9.9
Female % (n)	89 (72)
Caucasian % (n)	97 (79)
APS % (n)	10 (8)
Sjögren’s syndrome % (n)	5 (4)
Clinical characteristics at flare occurrence	
Anti-dsDNA % (n)	62 (49)
SLEDAI (mean±SD)	8.3±4.6
Hypocomplementaemia C3 % (n)	72.8 (59)
Hypocomplementaemia C4 % (n)	39.5 (32)
C BILAG C domain % (n)	5 (4)
Mucocutaneous BILAG domain % (n)	25 (20)
Muscculoskeletal BILAG domain % (n)	50 (40)
Cardiopulmonary BILAG domain % (n)	12 (10)
Haematological BILAG domain % (n)	6 (5)
Vascular BILAG domain % (n)	1 (1)
Neuropsychiatric BILAG domain % (n)	1 (1)
BILAG A % (n)	46 (37)
BILAG B % (n)	43 (35)
BILAG C % (n)	11 (9)
SLICC/DI >0% (n)	31 (24)
SLICC/DI median (IQR)	2 (1–2)
Ongoing treatments at flare occurrence	
IS therapy %	36 (29)
Belimumab % (n)	8 (7)
Azatioprine % (n)	6 (5)
Mycophenolate mofetil % (n)	7 (6)
Metotrexate % (n)	14 (11)
Cyclophosphamide % (n)	3 (2)
Ciclosporin % (n)	4 (3)
Leflunomide % (n)	2 (2)
Rituximab % (n)	1 (1)
Hydroxychloroquine % (n)	63 (51)
IS-naïve % (n)	27 (22)

APSantiphospholipid syndromeBILAGBritish Isles Lupus Assessment Groupdsdouble strandedGC, glucocorticoids; IS, immunosuppressantsSLEDAISLE Disease Activity IndexSLICC/DISystemic Lupus International Collaborating Clinics/Damage Index

Briefly, 72 flares (89%) occurred in female patients and 79 in Caucasians (97%). At the moment of the flare, the mean±SD age was 41.0±12.3 years, and the mean disease duration was 9.9±9.8 years.

The median SLEDAI score resulted 8 (5–10); according to the BILAG definitions, 40 flares (50%) were musculoskeletal, 20 (25%) mucocutaneous, 4 (5%) constitutional, 10 (12%) cardiopulmonary, 5 (6%) haematological, 1 vasculitic (1%) and 1 (1%) neuropsychiatric. Thirty-seven flares were categorised as ‘BILAG A’ (46%), 35 (43%) as ‘BILAG B’ and 9 (11%) as ‘BILAG C’.

At baseline, 25 flares (30%) had at least one item of organ damage according to SDI (median 2, IQR 1–2).

At the time of flare occurrence, 29 (36%) were on IS therapy, 52 were not taking IS drugs and among these 22 (27%) were IS-naïve. Details of the ongoing therapies are reported in table 1.

Treatments received to treat the disease flares are summarised in table 2.

**Table 2:** Treatments administered for disease flare

Therapy	% (n)
GC pulses	15 (12)
Oral GC	85 (69)
Median daily dose mg (median, IQR)	7.5 mg (5–10)
Introduction of hydroxychloroquine	8 (10)
Introduction of IS	78 (63)
Change IS	22 (18)
Mycophenolate mofetil	14 (11)
Cyclophosphamide	4 (3)
Rituximab	4 (3)
Azatioprine	6 (5)
Belimumab	58 (47)
Metotrexate	26 (21)
Ciclosporin	5 (4)
Leflunomide	2 (2)
Anifrolumab	2 (2)
Tacrolimus	1 (1)

GC, glucocorticoids; IS, immunosuppressants

Briefly, in most of the cases (63, 78%), a new IS (conventional or biological) was added on top of the background therapy, while in 18 cases (22%) there was a change in IS. In 47 cases (58%), a conventional IS was introduced, while in 52 cases (64%), a biological drug was introduced.

In 12 cases (15%), patients also received GC pulses, while 63 (78%) received oral GC therapy (median dose: 7.5 mg of PDN equivalent).

### Outcomes at 3, 6 and 12 months of follow-up

**Figure 1:**
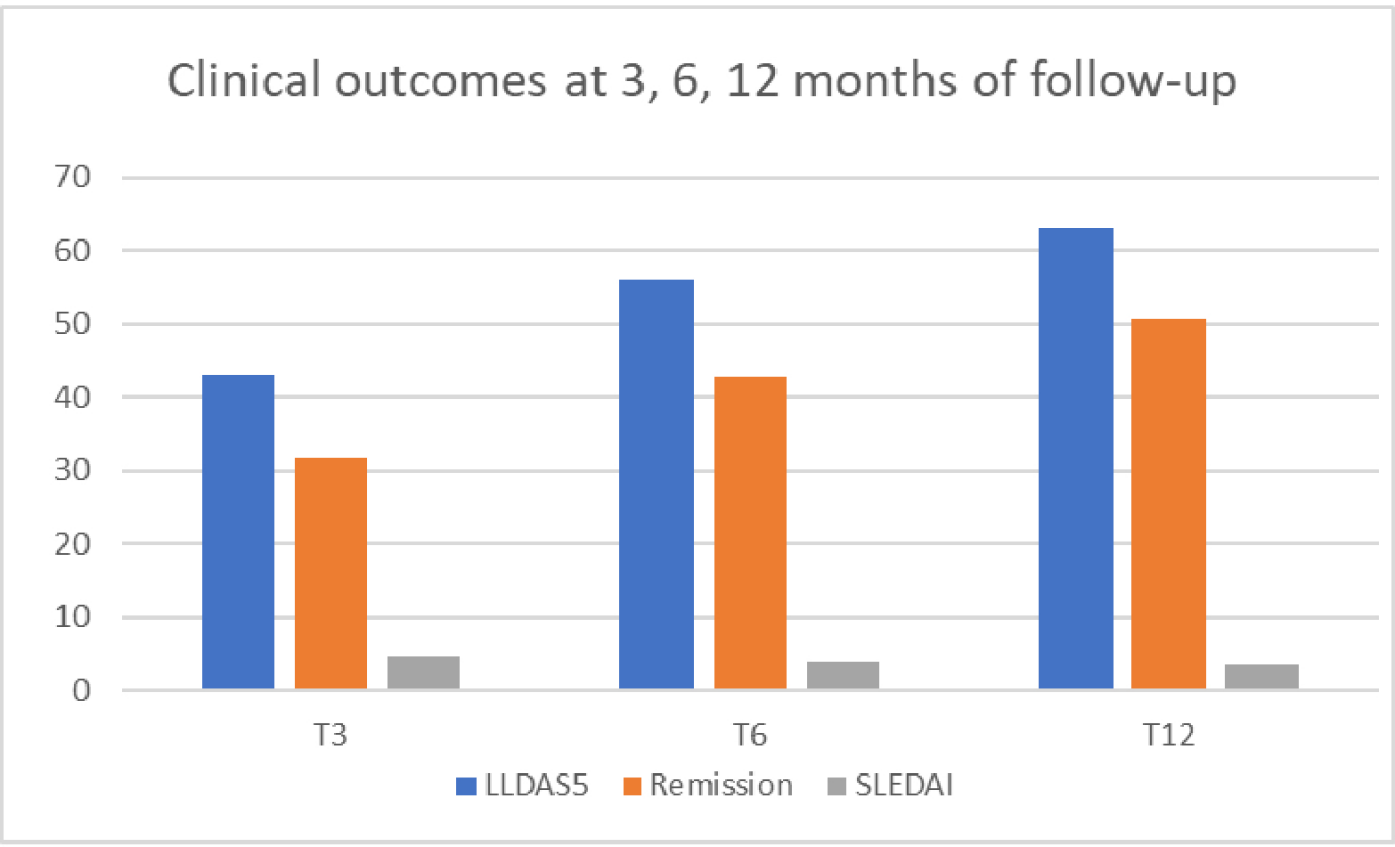
Clinical outcomes at 3, 6, 12 months of follow-up. LLDAS5, lupus low disease activity state; SLEDAI, SLE Disease Activity Index.

The detailed description of the clinical outcomes at the different time-points is reported in figure 1.

At 3 months of follow-up, 34 patients flaring (42%) were in LLDAS5 and 25 (31%) were in remission. At this time-point, the median SLEDAI resulted in 4 (IQR 2–5) and the median daily dosage of PDN equivalent resulted in 5 mg (IQR 5–8.75). Despite a significant SLEDAI reduction (mean at T0 of 8.3 vs mean at T3 of 4.8, p<0.01), only 39 patients (48%) satisfied the definition for SLEDAI improvement.

Among the 47 patients who were not in LLDAS5 at 3 months, 18 showed improvement to LLDAS.

In 5 cases (6%), treatment was discontinued thereafter because adverse events occurred between treatment onset and 3 months of follow-up. These cases were excluded from the subsequent analyses.

At 6 months, among the remaining 76 flares, 43 (56%) were in LLDAS5 and 32 (42%) were in remission; subsequently, at 12 months, 49 (64%) and 37 (49%) were respectively in LLDAS5 and remission. A graphic representation of the time required to remission and LLDAS5 is reported in online supplemental figures S1 and S2.

**Figure 2:**
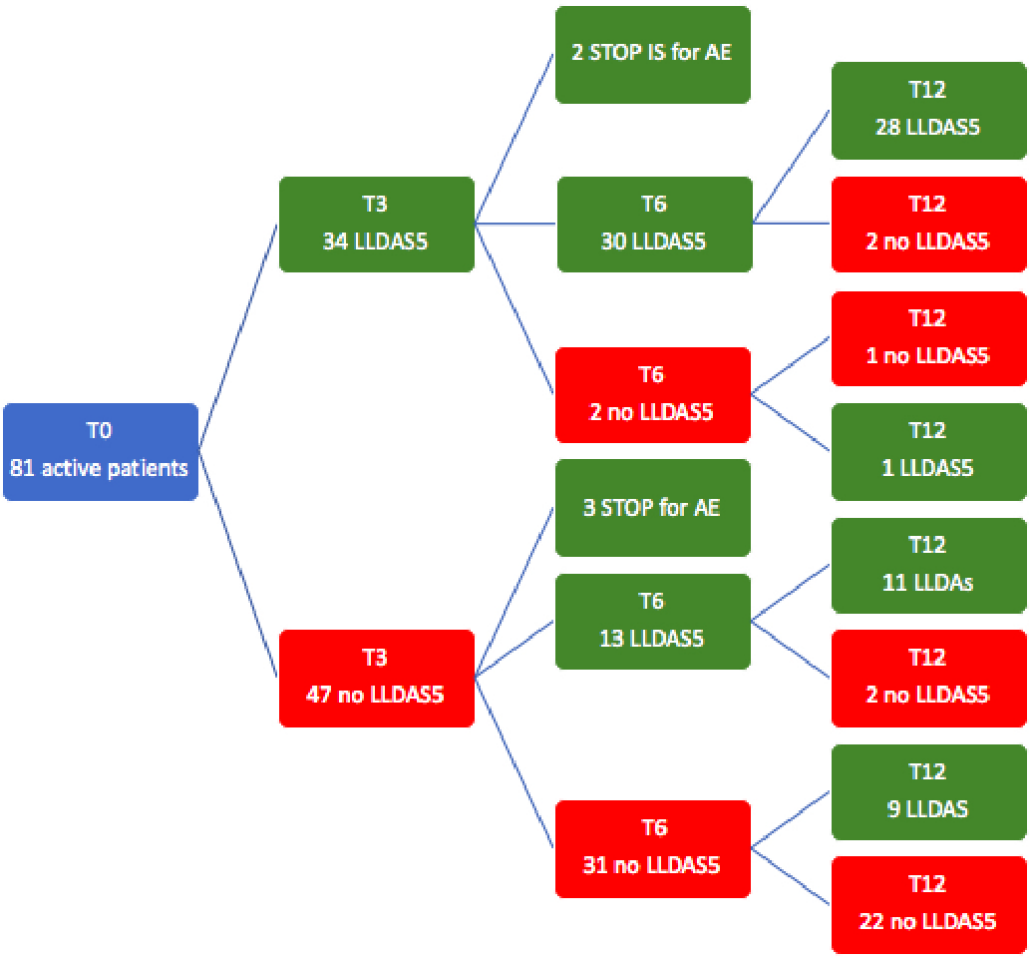
Disease state flow during the follow-up. AE, adverse event; IS, immunosuppressants; LLDAS5, lupus low disease activity state.

The disease state flow during the follow-up is also schematised in figure 2. Briefly, among patients who were in LLDAS5 at 12 months (n=49), 30 (61%) had already achieved LLDAS5 after 3 months of therapy (*early responders*); thereafter, among these, two patients lost LLDAS5 status at 6 months and one regained it at the end of follow-up while the other remained among non-responders at 12 months.

Among the remaining 20 patients in LLDAS5 at 12 months, 13 (65%) had already achieved LLDAS5 after 6 months of therapy.

Considering the entire cohort, only 9 patients (11%) required a longer time to respond and reached LLDAS5 status at 12 months, but not earlier (*late responders*).

Both ‘late responders’ and ‘early responders’ subgroups were not different in terms of demographic characteristics, baseline clinical data, GC dosage or type of IS treatment started (biological or not) with each other and with respect to the rest of the cohort (online supplemental table S1).

Cox model revealed a significant contribution of a change in IS (p=0.019), but not of age and disease duration on LLDAS5. No significant contribution of a modification in IS, age, disease duration on remission was found.

Contingency table analysis showed a significant relationship (p=0.004) between IS change at 6 months (19% of flares) and individuals in LLDAS5 at 12 months (63%). The association between the change in IS at 3 months (6%) and the percentage and individuals in LLDAS5 at 6 months (56%) was not significant (p=0.59) (online supplemental file 1).

Furthermore, when the analysis was repeated using remission as outcome, contingency table analysis showed a significant association (p=0.015) between IS change at 6 months (19% of flares) and remission rate at 12 months (51% of flares). The association between the change in IS at 3 months (7%) and the percentage of remissions at 6 months (42%) did not reach significance (p=0.07).

To evaluate the impact of the different types of therapy, we considered only flares on monotherapy (n=59) with conventional IS or with biological drugs. Concerning the association between the type of IS (conventional, biological) and the outcome, the results did not show a link at 3 months for remission or LLDAS5.

Conversely, at 6 months, the association was significant with LLDAS5 and showed a tendency for remission; indeed, 66% of the subjects in LLDAS5 took biological IS (vs 37% of patients not in LLDAS5, p=0.044). Likewise, 68% of the subjects in remission took biological IS (vs 42% of patients not in remission).

At 12 months of follow-up, a tendency of association was present for both LLDAS5 and remission. Specifically, 59% of the subjects in LLDAS5 took biological drugs (vs 30% of patients not in LLDAS5), and 52% of subjects in remission took biological drugs (vs 37%).

A qualitative representation of the specific type of drug and its association with disease status (LLDAS5 achieved or not) at different time-points is depicted in onlinesupplemental figures S3S5.

In order to evaluate if an early detection of non-responders could be possible, we analysed the proportion of patients ‘not improving’ in SLEDAI at 3 months (33 patients). As expected, the majority of these patients (23, 70%) were also not in LLDAS5 at 3 months. Not being in LLDAS5 at 3 months, nor having an improving SLEDAI score, was not associated with a poor outcome at 12 months. Thus, based on these data, it is not possible to estimate the long-term outcome based on the lack of early clinical improvement.

We did not find any association between being treated with GC pulses and the outcomes at 3, 6 and 12 months. Moreover, the use of GC pulses was not associated with early or late responses.

## Discussion

This study was aimed to investigate the time needed to achieve a clinical response following the initiation of a new IS drug in patients with SLE presenting an extrarenal flare.

We identified 81 patients flaring with extrarenal manifestations that required a treatment change (both conventional and biological IS drugs). Over a 12-month period following the introduction of a new therapy, we monitored the clinical trajectories of these flares. Our data reveal that 3 months after starting the new IS therapy, one-third of the flares were in remission, with approximately 40% achieving LLDAS5, regardless of whether conventional or biological IS was administered. These percentages increase over time, with 64% achieving LLDAS5 and 42% reaching remission at 6 months. By 12 months, the percentages were 60% for LLDAS5 and 48% for remission.

In a T2T approach, timely treatment adjustments are recommended once the desired goal is not met to prevent prolonged exposure to disease activity. However, in the treatment of extrarenal flares, the right time to change IS is still unclear.

In this study, we have identified the 3–6 months timeframe as the most advantageous for assessing treatment effectiveness. Indeed, at the 3-month time-point, only 43% and 31.6% of flares were respectively in LLDAS5 and remission, and a substantial proportion of patients reached the therapeutic targets thereafter (56% LLDAS5 and 43% remission at 6 months). Conversely, extending observation without treatment changes up to the 12-month time-point seems to result in a minimal increase in the percentage of patients reaching LLDAS5 or remission state, with only 12% of flares showing a late response; thus, the risk of undertreating patients after this period becomes preponderant.

Interestingly, biological therapies seem to be associated with an earlier attainment of disease targets compared with conventional IS drugs. This observation is based on preliminary data from a small subgroup of patients, and it should be validated in larger studies. If confirmed, these findings could add a new piece of knowledge on the role of biological treatments in the armamentarium of SLE. These results are in line with previous reports from similar recent cohorts.^19^

Similarly, a recent meta-analysis explored the time to response after starting belimumab in patients with cutaneous involvement in SLE or cutaneous lupus erythematosus; they found a first significant response at 20 weeks of treatment with a substantial maintaining of the result through 52 weeks.^20^

We acknowledge some limitations in this study. First of all, the relatively small sample size did not allow a subgroup analysis of therapy; it is plausible that some differences in the time to response could be attributed to the type of treatment initiated. For instance, among biologics, we could argue that belimumab and anifrolumab can have a very different time to response and putting together these drugs could be incorrect. However, this study was intended to provide a broad indication of the timeframe for expecting a clinical response before considering a change in therapeutic strategy. Certainly, within these general frameworks, individual patient response times may be influenced by several factors, including the specific therapy initiated and individual clinical variables that must be considered. Despite its size, the cohort analysed is homogeneous and very well characterised, deriving from a single referral centre for SLE.

Another limitation of the study relies on the fact that follow-up duration is limited to 12 months; thus, we cannot entirely rule out the possibility that some patients classified as ‘non-responders’ at 12 months might show a delayed response to treatment. For instance, late clinical improvements have been observed in studies of belimumab, even if this cannot be generalised. Extending follow-up beyond 12 months could lead to undertreatment of patients and expose them to the potential risk of damage accrual.^21^

Finally, the analysis did not take into account background GC therapy, which may have somehow influenced the time to response. For instance, in a recent study by Ruiz-Irastorza *et al*, the administration of GC pulses to active patients with SLE during the first year of the disease is associated with a higher frequency of prolonged remission.^22^

However, in this cohort only a minority of patients received pulsed GC and it can be assumed that the treatment approach with GC was quite homogeneous, since in our centre in the last years we used standardised low-dose GC protocols.

To the best of our knowledge, this is the first study aiming at establishing the time to treatment response in a real-life setting.

In conclusion, our findings demonstrate that in a considerable percentage of cases, extrarenal manifestations of the disease exhibit a rapid response to established therapy, with the first clinically significant target achieved within 3 months of treatment initiation. Moreover, clinical improvement occurs in the majority of cases within 6 months of therapy. A very delayed response (observed at 12 months after initiation of therapy) is also possible, but this occurs in a minority of patients.

Therefore, a 6-month period from the initiation of new therapy could represent the timing to evaluate the response in a T2T approach in SLE. Further prospective analyses involving larger cohorts are warranted to validate these observations.

## supplementary material

10.1136/lupus-2024-001207 online supplemental file 1

10.1136/lupus-2024-001207 online supplemental figure 1

10.1136/lupus-2024-001207 online supplemental figure 2

10.1136/lupus-2024-001207 online supplemental figure 3

10.1136/lupus-2024-001207 online supplemental figure 4

10.1136/lupus-2024-001207 online supplemental table 1

## Data Availability

Data are available on reasonable request.
