# Supplementary material for: When is the right time to change therapy? An observational study of the time to response to immunosuppressive drugs in systemic lupus erythematosus
Source: Lupus Sci Med. 2024 Jul 23;11(2):e001207. doi: 10.1136/lupus-2024-001207 (PMC11268067; doi:10.1136/lupus-2024-001207)
Supplement: online supplemental file 1 [file lupus-11-2-s001.pdf]

Supplementary file: Contingency tables

1)

IS at 3 Months	LLDAST5 at 6 months		
	No	In LLDAS	Total
No change	44%	56%	100%
	94%	95%	94%
Change	50%	50%	100%
	6%	5%	**6%**
Total	44%	**56%**	100%
**P = 0.59.**			

2)

IS at 6 Months	LLDAST5 at 12 months		
	No	In LLDAS	Total
No change	29%	71%	100%
	63%	91%	81%
Change	71%	29%	100%
	37%	9%	**19%**
Total	37%	**63%**	100%
**P = 0.004.**			

3)

IS at 3 Months	DORIS at 6 months		
	No	In DORIS	Total
No change	55%	45%	100%
	91%	100%	93%
Change	100%	0	100%
	9%	0	**7%**
Total	58%	**42%**	100%
**P = 0.07.**			

4)

IS at 6 Months	DORIS at 12 months		
	No	In DORIS	Total
No change	42%	58%	100%
	69%	92%	81%
Change	79%	21%	100%
	31%	8%	**19%**
Total	49%	**51%**	100%
**P = 0.015**			
