# Supplementary figures and images for: When is the right time to change therapy? An observational study of the time to response to immunosuppressive drugs in systemic lupus erythematosus
Source: Lupus Sci Med. 2024 Jul 23;11(2):e001207. doi: 10.1136/lupus-2024-001207 (PMC11268067; doi:10.1136/lupus-2024-001207)

Figure S1: time to DORIS remission

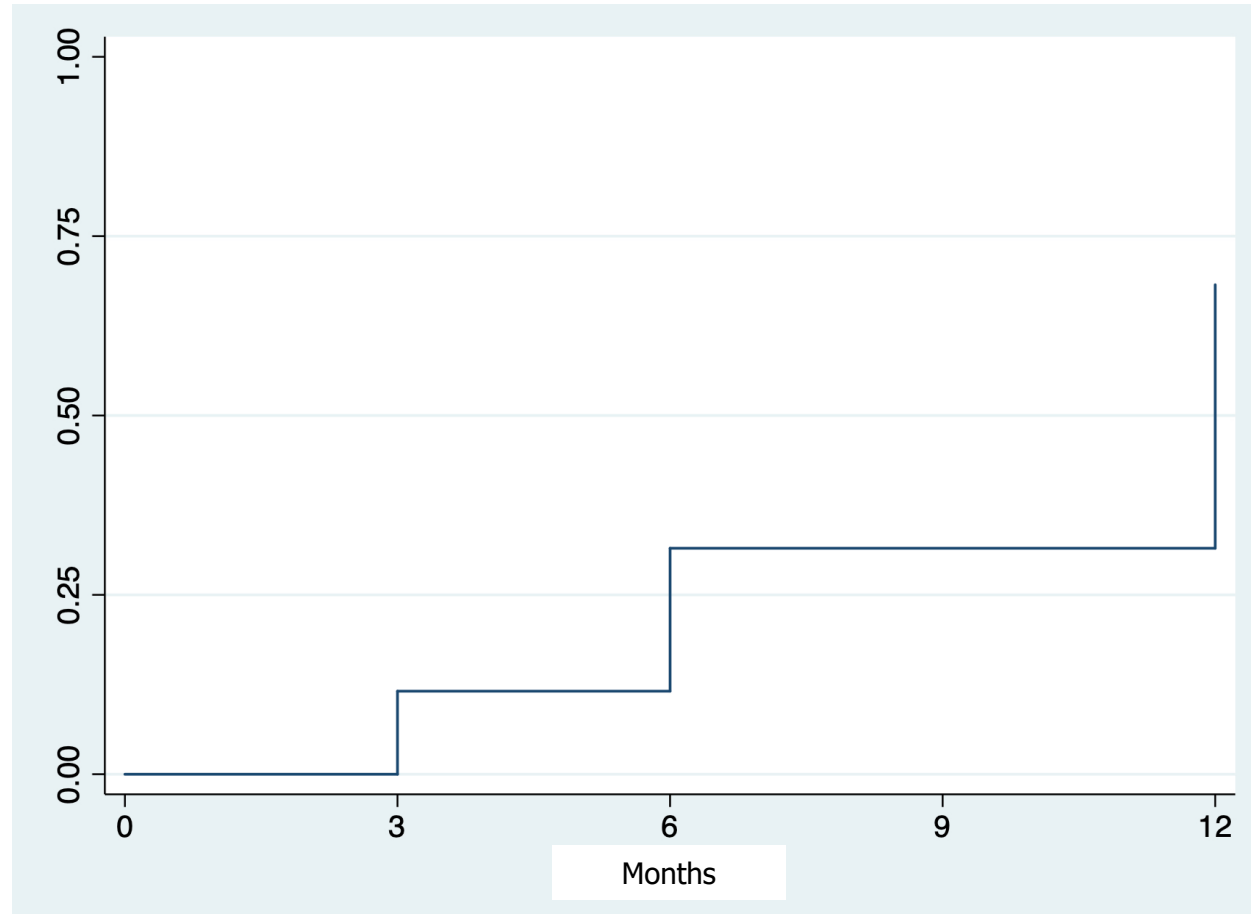

Figure S2: time to LLDAS5

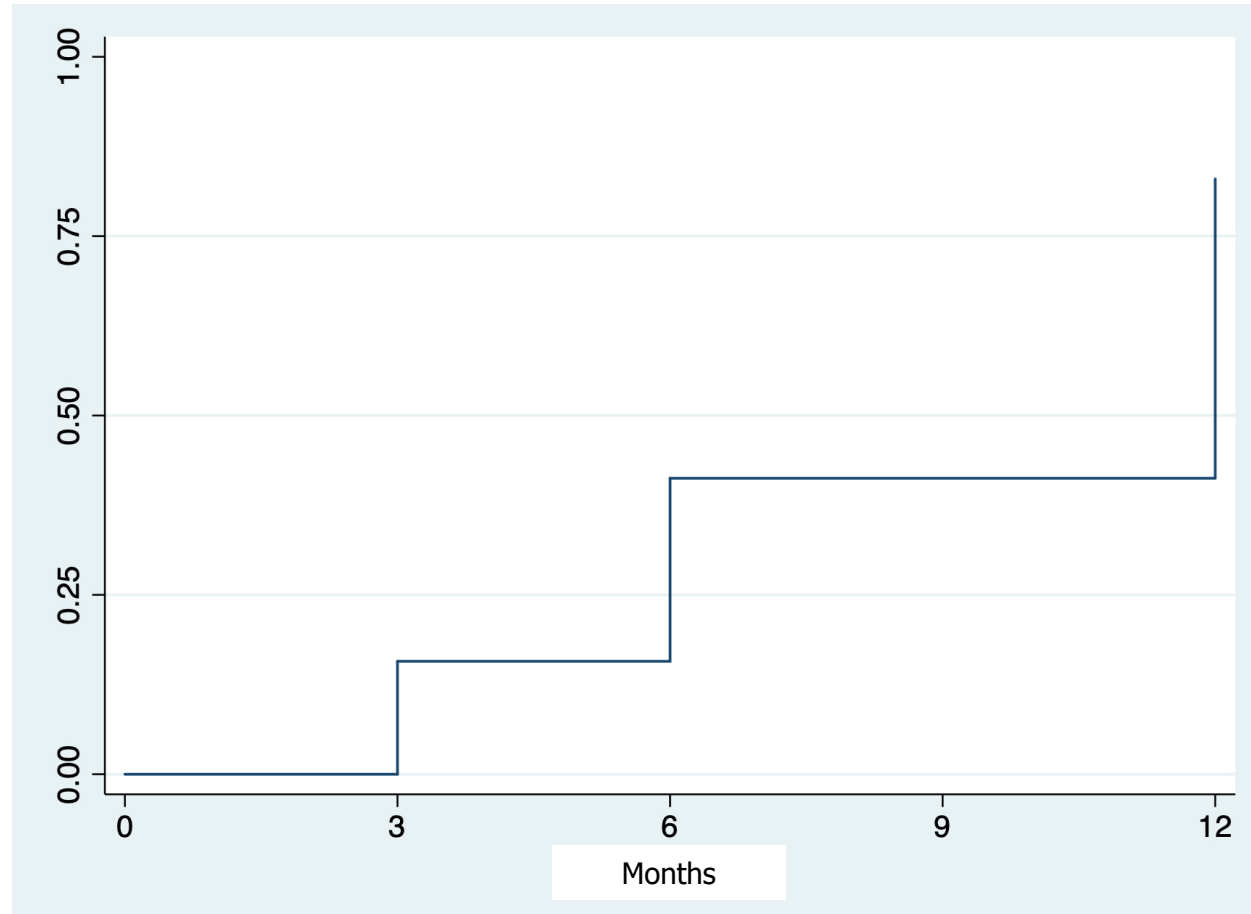

Supplement: online supplemental figure 1 [file lupus-11-2-s002.pdf]
