# Supplementary material for: When is the right time to change therapy? An observational study of the time to response to immunosuppressive drugs in systemic lupus erythematosus
Source: Lupus Sci Med. 2024 Jul 23;11(2):e001207. doi: 10.1136/lupus-2024-001207 (PMC11268067; doi:10.1136/lupus-2024-001207)
Supplement: online supplemental figure 4 [file lupus-11-2-s005.pdf]

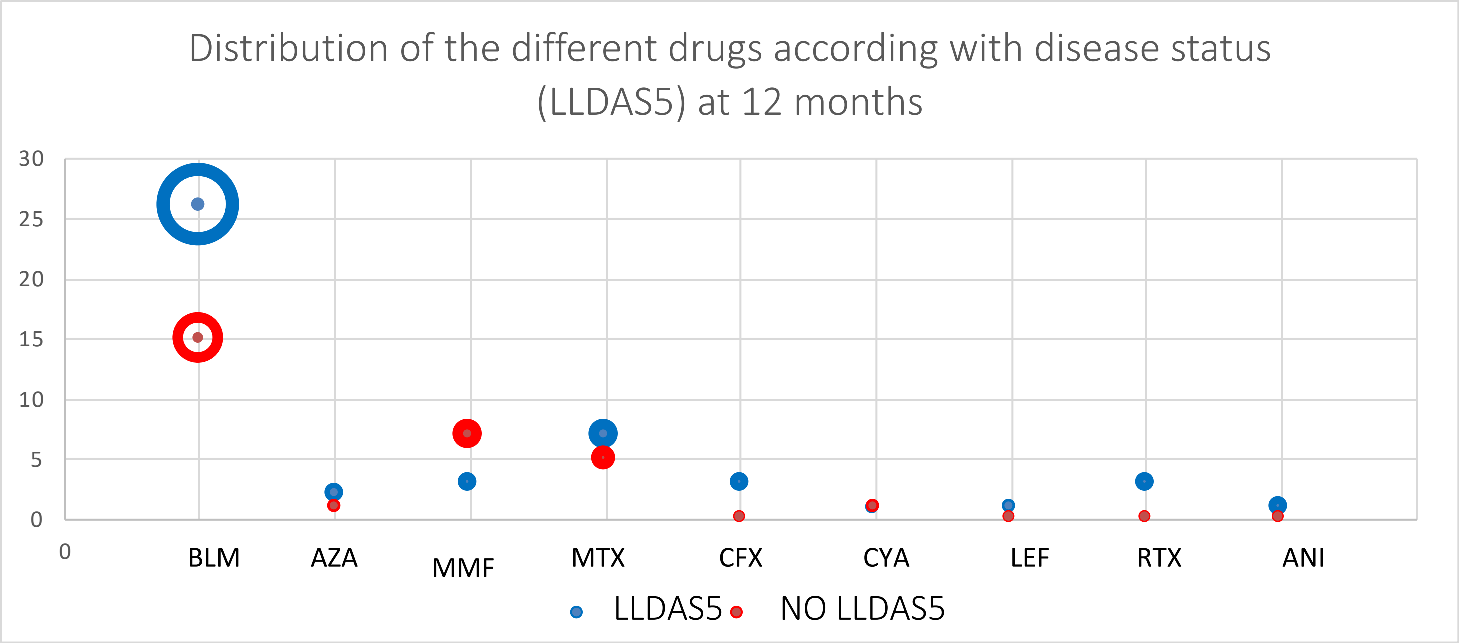

Legend:
BEL=Belimumab, AZA=Azathioprine, MMF= Mycophenolate Mophetile,
MTX= methotrexate; CFX=Cyclophosphamide
LEF= Leflunomide, RTX=Rituximab, ANI=Anifrolumab
Note: Dot size is proportional to the number of patients taking the drug
