# Supplementary material for: When is the right time to change therapy? An observational study of the time to response to immunosuppressive drugs in systemic lupus erythematosus
Source: Lupus Sci Med. 2024 Jul 23;11(2):e001207. doi: 10.1136/lupus-2024-001207 (PMC11268067; doi:10.1136/lupus-2024-001207)
Supplement: online supplemental table 1 [file lupus-11-2-s006.pdf]

Table S1: comparison of baseline characteristics between early responders, late responders and the rest of the cohort

	**Early responders N=30**	**Late responders N=9**	**Rest of the cohort N=37**	**p**
age (mean± SD)	40.3± 11.8	41.4± 12.4	41.1±13.2	n.s.
sex (F %)	83%	88%	91%	n.s.
disease duration (median-IQR)	7 (3-17)	3.5 (0.5-6.5)	7 (3-17)	n.s
SLEDAI_T0 (median-IQR)	6 (4-8)	8 (4-11)	8 (6-11)	n.s.
daily GC dose mg_T0 (median-IQR)	5 (5-10)	10 (5-15)	10 (5-37.5)	n.s.
IS naive (%)	33.3%	22.2%	24.3%	n.s.
BILAG A (%)<br>BILAG B (%)<br>BILAG C (%)	40%<br>43.3%<br>16.6%	66.6%<br>22.2%%<br>11.2%	40.5%<br>54.1%<br>5.4%	n.s.
